# Optimizing CT Abdomen–Pelvis Scan Radiation Dose: Examining the Role of Body Metrics (Waist Circumference, Hip Circumference, Abdominal Fat, and Body Mass Index) in Dose Efficiency

**DOI:** 10.3390/tomography10050049

**Published:** 2024-04-24

**Authors:** Huda I. Almohammed, Wiam Elshami, Zuhal Y. Hamd, Mohamed Abuzaid

**Affiliations:** 1 Department of Radiological Sciences, College of Health and Rehabilitation Sciences, Princess Nourah bint Abdulrahman University, P.O. Box 84428, Riyadh 11671, Saudi Arabia; 2 Medical Diagnostic Imaging Department, College of Health Sciences, University of Sharjah, Sharjah P.O Box 27272, United Arab Emirates; 3 Research Institute for Medical and Health Sciences, University of Sharjah, Sharjah P.O Box 27272, United Arab Emirates

**Keywords:** abdominopelvic CT scans, radiation dose, personalized dosimetry, optimization, body metrics, DLP, CTDI_vol_, SSDE, BMI

## Abstract

**Objective**: This study investigates the correlation between patient body metrics and radiation dose in abdominopelvic CT scans, aiming to identify significant predictors of radiation exposure. **Methods:** Employing a cross-sectional analysis of patient data, including BMI, abdominal fat, waist, abdomen, and hip circumference, we analyzed their relationship with the following dose metrics: the CTDI_vol_, DLP, and SSDE. **Results:** Results from the analysis of various body measurements revealed that BMI, abdominal fat, and waist circumference are strongly correlated with increased radiation doses. Notably, the SSDE, as a more patient-centric dose metric, showed significant positive correlations, especially with waist circumference, suggesting its potential as a key predictor for optimizing radiation doses. **Conclusions:** The findings suggest that incorporating patient-specific body metrics into CT dosimetry could enhance personalized care and radiation safety. Conclusively, this study highlights the necessity for tailored imaging protocols based on individual body metrics to optimize radiation exposure, encouraging further research into predictive models and the integration of these metrics into clinical practice for improved patient management.

## 1. Introduction

Computed Tomography (CT) has revolutionized medical diagnostics, including abdominal and pelvic imaging. Its ability to provide detailed cross-sectional views of internal organs has made it an invaluable tool for diagnosing and managing various diseases. The evolution of CT technology has been marked by significant advancements, from developing multi-detector CT scanners to integrating sophisticated software that enhances image quality and reduces motion artifacts. Despite these advancements, CT scanning raises critical concerns regarding patients’ radiation doses [1,2].

The worry about radiation exposure from CT scans is well founded. Ionizing radiation, employed in CT scans, has been associated with a marginal elevation in cancer risk. This risk is of particular concern in patients who require multiple scans, such as those with chronic conditions or in follow-up care. The researcher and scientists have thus been vigilant about minimizing unnecessary exposure and optimizing the radiation dose used in CT scans. This concern has led to the development of various dose optimization strategies, including using lower radiation doses for imaging without compromising the diagnostic quality of the images [3,4].

Personalized dosimetry in CT imaging is an area of growing interest and research. The concept revolves around tailoring the radiation dose to the individual patient’s body composition to ensure the lowest possible dose is used while still achieving high-quality diagnostic images. This approach is particularly relevant in abdominal and pelvic CT scans, where the body size and composition variability can significantly impact the radiation dose needed for optimal imaging [1,5].

Body metrics such as waist circumference (WC), hip circumference (HC), body mass index (BMI), and abdominal fat have been identified as potential factors that could influence the radiation dose in CT scans. These metrics provide a more individualized assessment of a patient’s body composition than traditional methods, which rely on standardized phantoms. Abdominal, waist, and hip circumference are essential because they directly affect how radiation is absorbed and scattered within the body. BMI, which considers both weight and height, is another crucial factor that could influence radiation dose requirements. The rationale is that these metrics, when considered, could lead to more precise and optimized dosing, potentially reducing the risk of overexposure in larger patients or underexposure in more minor patients [6,7,8].

Fat distribution around the abdomen and pelvis is another area of interest. It is hypothesized that the amount and distribution of trunk fat could influence the quality of CT images and the required radiation dose. This is because fat attenuates radiation differently from other tissues, which could necessitate adjustments in the radiation dose for optimal imaging [9,10,11].

### 1.1. Gaps in Current Research and Knowledge

Despite the recognized importance of these body metrics in dose optimization, there remains a significant gap in the current research. Most studies have focused on the automated collection of dose data and the detection of dose outliers, with less emphasis on the direct correlation between body metrics and dose optimization. Additionally, there is a lack of comprehensive data on how different body metrics, when considered together, impact the radiation dose in CT scans.

### 1.2. Study Aim and Objectives

This study comprehensively analyzes the relationships between BMI, abdomen, waist, and hip circumference, and abdomen fat (AF) and their collective impact on radiation dose during abdominal and pelvic CT scans. The objective is to identify critical variables that significantly influence radiation dosing effectiveness and to develop a model that can be used to optimize radiation doses in clinical practice. The goal is to enhance patient safety by minimizing radiation exposure while maintaining the diagnostic quality of CT images.

### 1.3. The Relevance and Significance of this Study

The findings of this study have the potential to impact clinical practice significantly. By providing a more nuanced understanding of how different body metrics influence radiation dose, this study could lead to the development of more personalized imaging protocols. These protocols would improve patient safety by reducing unnecessary radiation exposure and ensuring that the diagnostic quality of the images is not compromised. In the broader context, this study contributes to the field of personalized medicine, where treatments and diagnostics are tailored to individual patient characteristics, thereby enhancing the overall quality of healthcare.

## 2. Material and Methods

### 2.1. Study Design and Data Collection

This study was designed as a cross-sectional prospective analysis of CT abdomen–pelvis scans. The population sample included 61 patients, randomly selected from the patients who underwent abdomen–pelvis scans without contrast CT scans for various indications such as kidney stones, chronic liver diseases, appendicitis, pancreatitis, assessing bowel obstructions, and unexplained abdominal pain. The inclusion criteria were adult patients (age ≥ 18 years), with both emergency and elective scans considered, who agreed to participate in this study during the data collection period. Exclusion criteria included patients with incomplete data records and those who underwent procedures that deviated from the standard scan protocol. This standard protocol specifies predefined settings for slice thickness, tube voltage, current, and contrast use, optimized for abdominopelvic imaging. Deviations might involve adjustments like increased tube voltage or altered contrast timing to accommodate specific patient conditions or clinical needs, which could affect radiation doses and image consistency across this study. The patient’s ages range between 18 and 80 years, with a mean of 39.6 and a standard deviation of 15.6.

### 2.2. CT Machine

A high-definition CT scanner, essential for detailed diagnostic imaging, was used to ensure the acquisition of high-quality images necessary for accurate medical diagnosis.

### 2.3. Scan Protocol

The scan protocol involved a standardized abdomen–pelvis CT examination. Scans were performed with the patients supine, using predefined parameters optimized for abdominal and pelvic imaging. The protocol was designed to balance image quality with radiation dose minimization.

### 2.4. Body Measurements (BMI, AC, HC, WC, APD, LD, and AF)

Patient body measurements encompass body mass index (BMI), abdomen (AC), waist (WC), and hip (HC) circumference, and abdominal fat (AF). BMI was calculated using the patient’s weight in kilograms divided by their height in square meters. AC, WC, and HC were obtained manually through established anthropometric methods. AC was measured at the largest abdominal area, WC was measured at the umbilicus level, and HC was taken at the hips’ widest point. AF was gauged at the abdomen’s widest part, extending from the skin to the abdominal muscles [11,12,13,14]. These dimensions were precisely measured by a trained staff member who collected the data from all patients, ensuring accuracy and consistency.

### 2.5. Radiation Dose (CTDI_vol_, DLP, and SSDE)

Radiation dose metrics were extracted for each CT scan, including the Computed Tomography Dose Index (CTDI_vol_), Dose–Length Product (DLP), and Size-Specific Dose Estimates (SSDEs). The CTDI_vol_ and DLP are standard dose metrics the CT scanner provides, representing the radiation exposure per scan. The SSDE was calculated to account for patient size, providing a more personalized radiation dose estimate.

### 2.6. Data Analysis and Machine Learning

In this study, we employed ColabWithMe, a comprehensive analytical tool, to investigate the correlation between body measurements and radiation doses. The dataset underwent preliminary cleaning and preparation, including missing value checks and BMI calculation. Extensive exploratory data analysis (EDA) was performed using ColabWithMe to examine distributions and relationships between variables through histograms and a correlation heatmap. We conducted linear regression analyses to assess the relationship between body measurements (WC, HC, BMI, trunk fat) and radiation doses, thereby understanding their influence on the CTDI_vol_, DLP, and SSDE.

### 2.7. Ethical Approval

This research received approval from the University of Sharjah Research Ethics Committee under reference number 18-03-11-01, ensuring adherence to ethical standards in conducting this study. All methods and protocols were rigorously developed to align with the applicable guidelines and regulations. This demonstrates the commitment to ethical research practices and the integrity of this study’s design and execution. We obtained written informed consent from all participants before their inclusion in this study. This consent covered the use of their medical data for research purposes, with assurances of confidentiality and anonymity maintained throughout the analysis.

## 3. Results

### Descriptive Analysis

The descriptive analysis of the dataset provides a comprehensive overview of the distribution and central tendency of the radiation doses and body measurements collected during abdominal and pelvic CT scans.

– CTDI_vol_ (mGy): The average CTDI_vol_ is approximately 12.8, with a standard deviation (SD) of 3.8, indicating some variability in the dose index across the sample. The values range from a minimum of 7.45 to a maximum of 20.
– DLP (mGy-cm): The DLP has an average of 629.2, with a higher SD of 207.4, suggesting a wider spread of values. The DLP spans from 327 to 998.
– SSDE (mGy): The SSDE has a mean value of 25.04 and an SD of 3.67. Its values range from 22.2 to 35, showing moderate variability.
– BMI: BMI averages 29.1, with a substantial SD of 8.2, reflecting the diverse body compositions of the patients. BMI values range broadly from 19.8 to 57.9.
– Abdominal fat (AF) (cm): On average, abdominal fat measures 5.91 with a standard deviation of 1.50. The measurements vary from 3.2 to 9.4.
– Waist circumference (WC) (cm): The average WC is 96.09 cm, and the standard deviation is 13.31 cm, indicating some variability in this measurement. The range is from 73.7 cm to 121.9 cm.
– Abdomen circumference (Abd. C) (cm): The average (Abd. C) is 92.10 cm with a standard deviation of 13.65 cm, indicating a diverse set of measurements from 71.1 cm to 120 cm.
– Hip circumference (H.C) (cm) has a mean of 108.48 cm and a standard deviation of 12.79 cm, ranging from 88.9 cm to 134.6 cm.

**Figure 1:**
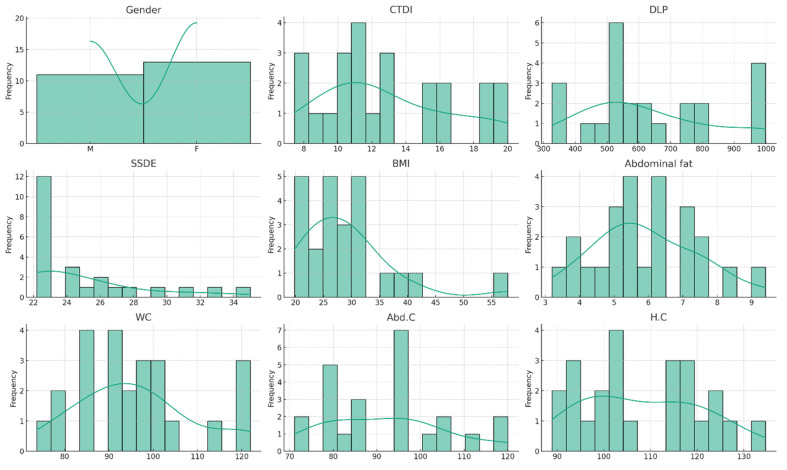
Histogram of body measurement and radiation dose.

The histograms (Figure 1) provide a visual representation of the data distribution for each variable. Most distributions appear roughly symmetrical, but some show signs of skewness. For instance, the BMI histogram indicates a right-skewed distribution, suggesting a subset of patients with a BMI significantly higher than the average.

**Figure 2:**
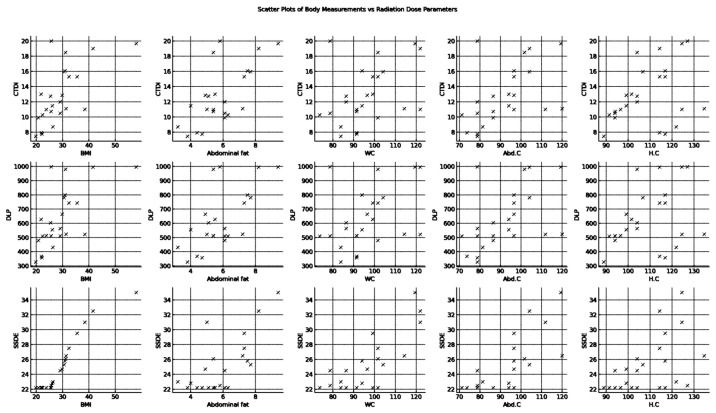
Scatter plots of body measurements vs. radiation dose parameters.

Figure 2’s scatter plots graphically depict the association between body measurements—BMI, AF, WC, AC, and HC—and radiation doses—the CTDI_vol_, DLP, and SSDE—in CT scans. The visual data suggest BMI, AF, and WC significantly correlate with the CTDI_vol_ and DLP, as evidenced by the rising trend lines correlating with body measurement increments. AC and HC demonstrate a weaker, statistically insignificant connection with these radiation doses. SSDE’s relationship with body measurements appears more complex, possibly non-linear, as data points cluster at lower SSDE values, and the relationship’s strength diminishes past certain measurement thresholds. These plots suggest BMI and AF notably affect the CTDI_vol_ and DLP, potentially due to how body mass and fat influence radiation penetration for imaging clarity. However, SSDE’s plot implies additional factors influence this dose parameter. Collectively, these plots underscore the utility of exploratory data analysis and signal the need for further statistical investigation to gauge the observed relationships’ strength and significance precisely.

**Figure 3:**
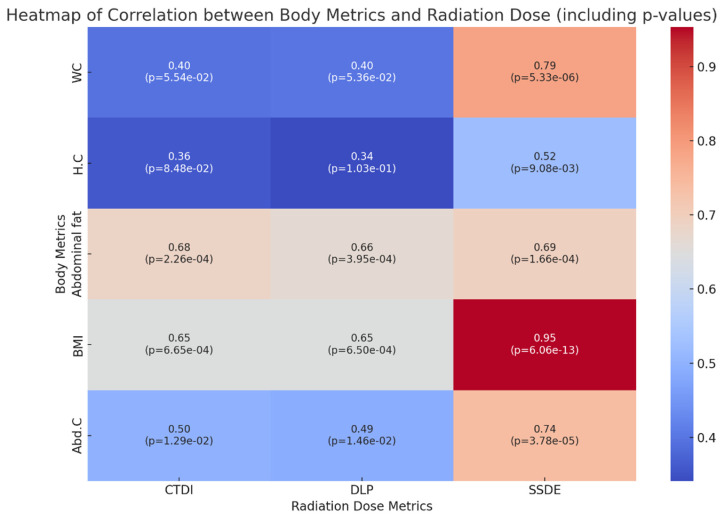
Correlation between body measurements and radiation dose.

Figure 3, the heatmap, was created using Pearson correlation coefficients to visualize the interrelationships between body measurements and radiation dose parameters from CT scans, offering a clear and concise overview of these associations. The enhanced heatmap integrates both the correlation coefficients and their corresponding *p*-values, offering a detailed visualization of the statistical relationships between various body metrics and radiation dose metrics in CT abdomen–pelvis scans. Each cell in the heatmap displays two key pieces of information: the Pearson correlation coefficient (upper value), indicating the strength and direction of the relationship, and the *p*-value (lower value), assessing the statistical significance of this correlation. The color intensity reflects the magnitude of the correlation, with warmer tones indicating stronger positive correlations. Significant findings include the exceptionally strong and statistically significant correlation between BMI and all radiation dose metrics, especially the SSDE (correlation coefficient of 0.954, *p*-value of 6.06 × 10^−13^), underscoring BMI’s critical influence on radiation dose efficiency. WC and Abd.C also demonstrate notable correlations with SSDEs, reinforcing their importance in dose determination. The inclusion of *p*-values, particularly those below the threshold of 0.05, confirms the statistical significance of these relationships, bolstering the heatmap’s value as a tool for informing more personalized and safer CT scanning protocols based on patient-specific body metrics.

## 4. Discussion

Patient factors significantly dictate the absorbed radiation dose and CT scanner output, highlighting the inadequacy of standard AAPM phantoms in capturing the actual variance in body size and composition across the patient population [15]. This discrepancy leads to the CTDI_vol_ measurement, as currently utilized in CT scanners, substantially underestimating the actual radiation dose to which patients are exposed. The American Association of Physicists in Medicine (AAPM) has proposed the use of the effective diameter (d_eff_) and the water equivalent diameter (d_w_) as more accurate metrics to assess patient size by accounting for body composition and attenuation. Despite d_w_ being hailed as the gold standard for its adaptability across diverse body sizes and tissue heterogeneity, its practical application is hampered by the laborious and time-consuming calculation process and the lack of immediate availability in commercial software globally. This gap underscores the pressing need for alternative patient body measurements that can be reliable predictors of the radiation dose encountered during CT examinations. By identifying such metrics, clinical practitioners could better gauge radiation dose trends relative to body size, thereby enhancing their ability to tailor radiation doses to individual patients, ensuring efficacy and safety in diagnostic imaging practices [15].

This study seeks to elucidate the correlation between patient body measurements and radiation dose, aiming to simplify the understanding of how patient body size collectively influences radiation exposure and to identify the most significant predictors of radiation dose in patients undergoing CT scans by examining the relationship between five physical measurement-based indices—BMI, abdominal fat, waist, abdominal, and hip circumference—that can be readily acquired from the patient. This study provides valuable insights into the potential for these body metrics to serve as proxies for more complex measurements, like the water equivalent diameter (d_w_). The inclusion of the SSDE as a key dose metric is particularly noteworthy, as it offers a mean dose value for the central image of the scan range, thereby presenting a practical and relevant measure for clinical users. By leveraging the d_w_ in calculating the SSDE, this study underscores its commitment to integrating established scientific methodologies with the practical needs of clinical practice, aiming to enhance patient care through optimized radiation dose management [10,11,12,13].

In our study, the observed values for the CTDI_vol_ (12.84 ± 3.81 mGy) and the SSDE (25.04 ± 3.67 mGy) exceeded those reported in a retrospective Chinese study [16], which noted the CTDI_vol_ at 9.66 ± 2.21 mGy and the SSDE at 13.72 ± 1.83 mGy. Similarly, our research’s mean (DLP) (629.29 ± 207.40 mGy.cm) surpassed findings from McLaughlin et al., who presented a DLP of 524 ± 236 mGy-cm [10]. Remarkably, the CTDI_vol_ from our analysis is below the achievable dose of 17 mGy identified in a 2020 United Arab Emirates (UAE) nationwide dose survey, which marked a reduction from 17 mGy to 12.84 mGy. Conversely, our DLP findings (629 mGy-cm) exceed the achievable dose of 605 mGy-cm reported in the same UAE study. Nonetheless, our recorded measurements significantly fall below the initial Diagnostic Reference Levels (DRLs) recommended by the Ministry of Health and Prevention (MOHAP) hospitals, where the CTDI_vol_ was suggested at 20 mGy and the DLP at 1025 mGy-cm [17]. This comparison highlights the variability in radiation dose metrics across different regions and studies, underscoring the importance of context-specific benchmarks in radiation dose management.

The CTDI_vol_, DLP, and SSDE increased with increasing BMI, abdominal fat, and waist circumference, which was made evident in successful regression plots displaying an upward trend. The correlation between body size metrics and the DLP and CTDI_vol_ in our study is consistent with the results of the retrospective study by Inoue et al., including data from over 3200 CT scans [18]. The positive correlation between BMI and CTDI_vol_ reported by Inoue et al. (0.88) was stronger than the findings of our study (0.65) and that of McLaughlin et al. (0.79) [10]. Again, a strong positive correlation between the DLP and BMI was reported by Inoue et al. (r = 0.86), whereas our study (r = 0.65) corroborated these findings. It is noteworthy that a study conducted in the UAE in 2020 also concluded that there was a positive correlation between the weight of the patient and the CTDI_vol_ (0.66) and the DLP (0.22) [19].

Another study that assessed data from preoperative liver CT examinations [11] noted a positive correlation between effective dose and abdominal fat. McLaughlin et al. also noted this correlation, indicating the predictability of abdominal adiposity and DLP. Our study yielded congruent results, with statistically significant strong positive correlations between the DLP, CTDI_vol_, SSDE, and abdominal fat.

Although waist, abdominal, and hip circumference correlated, a stronger positive correlation was lacking between the hip circumference and the dose metrics. The strongest significant correlation was found between the SSDE and waist circumference (0.79) and abdominal circumference (0.79). Therefore, our study contributes further to the established knowledge that patients with an increased cross-sectional area receive high radiation doses during abdominopelvic CT. Our study establishes that of hip, abdominal, and waist circumference, it is a patient’s waist circumference that is a stronger predictor of radiation dose during abdominopelvic CT examinations. The consistency of our study findings with other studies supports our conclusion that body size indices can inform radiation dose management in adult CT populations when accounted for as covariates.

The substantial correlation observed between BMI and the SSDE in our study is indicative of the fundamental principles underpinning the SSDE’s formulation, which inherently accounts for patient size. The SSDE adjusts for the attenuation properties of different body sizes, providing a dose estimate tailored to patient dimensions. Our research extends this concept by systematically analyzing the interplay between a spectrum of body measurements, including BMI and the SSDE. This approach not only reaffirms the SSDE’s critical role in reflecting the dose received by patients of varying body sizes but also contextualizes it within a broader framework of patient-specific dosimetry. This study illuminates the multifaceted nature of radiation absorption and scattering influenced by body metrics beyond BMI, such as abdominal fat and waist circumference, thereby offering a more comprehensive strategy for personalized radiation dose optimization in clinical radiology.

A strength of this research lies in its comprehensive analysis of how multiple body metrics collectively impact radiation exposure when modeled together. The methods and results have practical applications—by characterizing dose dependencies on measurable indices, like waist circumference, BMI, and abdominal fat, our work supports the development of more advanced personalized imaging protocols. This would enhance both patient safety through optimized radiation exposure and healthcare quality by maintaining diagnostic standards, even in high-risk populations. Overall, with larger validations, the approach of individually tailoring CT protocols based on readily collected anthropometric data holds promise for advancing precision medicine approaches in abdominal imaging.

## 5. Conclusions

This study has established a significant positive correlation between patient body metrics—specifically BMI, abdominal fat, and waist circumference—and radiation dose metrics (DLP, CTDI_vol_, SSDE) in abdominopelvic CT examinations. These findings underscore the impact of patient body size and composition on radiation exposure, reinforcing the need for personalized dosimetry in CT imaging to optimize radiation doses and enhance patient safety without compromising diagnostic quality.

Clinical protocols should be refined to incorporate patient-specific body metrics into dosimetry calculations, potentially through the development of automated software that can quickly assess and adjust doses. Further research should explore the integration of additional body metrics and their composite indices to refine dose optimization strategies. Large-scale, multicentred studies are recommended to validate these findings across diverse populations and scanner types, enhancing the generalizability and applicability of dose optimization protocols. Additionally, longitudinal studies examining the long-term outcomes of optimized dosing protocols on patient health and diagnostic efficacy would be invaluable. Investigating the application of artificial intelligence and machine learning in automating and refining dose adjustments based on body metrics could revolutionize personalized dosimetry in CT imaging.

## Data Availability

Data will be available upon request from the corresponding author.

## Abbreviations

CT	Computed Tomography
WC	Waist Circumference
HC	Hip Circumference
BMI	Body Mass Index
AF	Abdomen Fat
AC	Abdomen Circumference
CTDI_vol_	Computed Tomography Dose Index
DLP	Dose–Length Product
SSDE	Size-Specific Dose Estimate
Eda	Exploratory Data Analysis
SD	Standard Deviation
AAPM	American Association of Physicists in Medicine
UAE	United Arab Emirates
DRLs	Diagnostic Reference Levels
MOHAP	Ministry of Health and Prevention
